# Normal-Flow, Low-Gradient Severe Aortic Stenosis Quality of Life Improvements With TAVR: More Patients to Help?∗

**DOI:** 10.1016/j.jacadv.2023.100640

**Published:** 2023-10-05

**Authors:** Kayla A. Riggs, Megan M. McLaughlin, Amit Goyal

**Affiliations:** a Division of Cardiology, Department of Internal Medicine, University of Texas Southwestern Medical Center, Dallas, Texas, USA; b Division of Cardiology, Department of Internal Medicine, University of California-San Francisco, San Francisco, California, USA; c Division of Cardiology, Department of Internal Medicine, Veterans Affairs North Texas Health Care System, Dallas, Texas, USA

**Keywords:** moderate aortic stenosis, transcatheter aortic valve replacement, valvular disease

**Figure:**
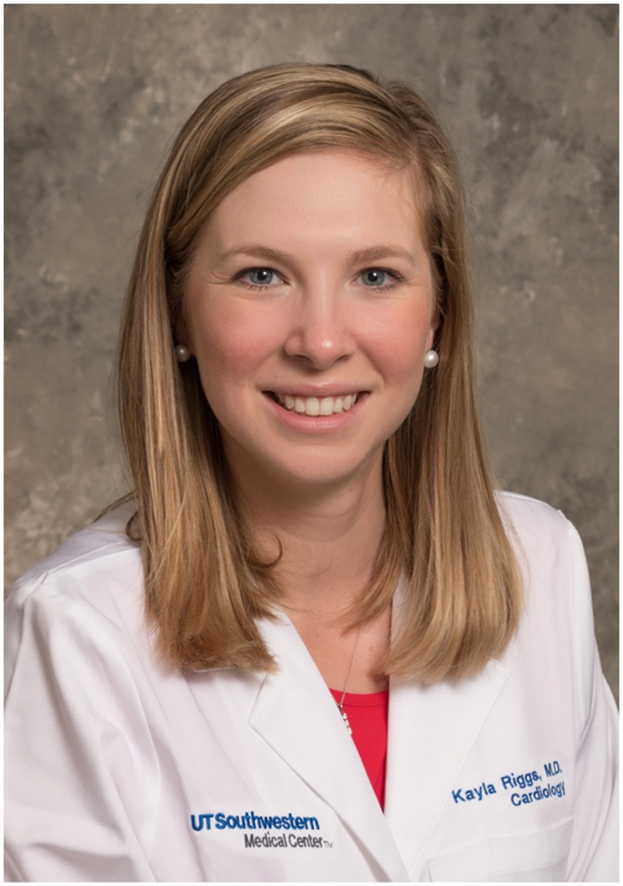

When treatment options are limited for a given pathology, we often underdiagnose and underappreciate its impact. Instead, we tend to only recognize its most obvious and clinically catastrophic manifestations. For example, the advent of tafamidis changed the landscape of cardiac amyloidosis from a once “rare” disease, driving identification of milder and even subclinical forms that were previously overlooked.^1^ Similarly, the advent of transcatheter therapies is revolutionizing our understanding of aortic stenosis (AS). When surgical aortic valve replacement was the only option, treatment was reserved for classic symptomatic severe high gradient AS (HG-AS) with aortic valve area (AVA) <1 cm^2^, mean gradient (MG) ≥40 mm Hg, and peak velocity (PV) ≥4 m/s. This began to change when transthoracic aortic valve replacement (TAVR) was found to be superior to medical therapy for inoperable severe AS.^2^ Subsequently, studies across the entire surgical risk spectrum found TAVR to be noninferior or superior to surgical aortic valve replacement, leading to Food and Drug Administration approval for TAVR for severe AS to include all surgical risk profiles in 2019.^3^, ^4^, ^5^, ^6^, ^7^, ^8^

Now, TAVR availability is driving increasing recognition of a variety of AS hemodynamic profiles with adverse prognostic implications—including low-flow low-gradient (LF-LG) and paradoxical LF-LG AS, as well as normal-flow, low-gradient (NF-LG) AS. The 2020 multisociety American guidelines designate a class 1 indication for intervention for symptomatic severe LF-LG and paradoxical LF-LG AS.^9^ The 2021 European guidelines also recommend consideration of intervention for symptomatic severe LF-LG AS but recommend surveillance for NF-LG AS due to reports indicating prognosis comparable with moderate AS.^10^ Data supporting intervention in NF-LG AS is conflicting. A comparison of over 400 asymptomatic patients, almost half with NF-LG AS, had similar outcomes to patients with moderate AS.^11^ In observational studies of patients with NF-LG, mortality was similar to moderate AS, with no significant difference observed in outcomes with intervention vs medical management.^12^, ^13^, ^14^ However, a meta-analysis revealed similar mortality rates between NF-LG AS and HG-AS, as well as improved outcomes with aortic valve replacement for patients with NF-LG AS.^15^ Research to better understand the natural history and prognosis of NF-LG AS is needed to guide therapeutic decision-making.

In this issue of *JACC: Advances*, Khaleel et al^16^ explored changes in quality of life (QoL) and survival in patients with NF-LG AS compared to HG-AS in a single-center retrospective study of 860 patients undergoing TAVR from 2013 to 2021. The patients were stratified into 4 AS hemodynamic profiles by flow state and aortic valve gradient: HG-AS (PV ≥4 m/s or MG ≥40 mm Hg regardless of left ventricular ejection fraction (LVEF) or flow state; 42.8% of patients); classical LF-LG AS (PV <4.0 m/s and MG <40 mm Hg, AVA <1.0 cm^2^, LVEF <50%; 8.6% of patients); paradoxical LF-LG AS (PV <4.0 m/s and MG <40 mm Hg, AVA <1.0 cm^2^, LVEF ≥50%, indexed stroke volume [SVi] <35 mL/m^2^; 20.1% of patients); and NF-LG AS (PV <4.0 m/s and MG <40 mm Hg, AVA <1.0 cm^2^, LVEF ≥50%, SVi ≥35 mL/m^2^; 28.5% of patients). The proportion of patients with NF-LG AS is itself notable and indicates the prevalence of this important subset being referred for TAVR.

All groups experienced significant improvements in baseline to 1-year Kansas City Cardiomyopathy Questionnaire (KCCQ) scores in both adjusted and unadjusted analyses, with similar proportions of patients achieving large improvements in QoL in the NF-LG AS and HG-AS groups (median increase of 20.8 vs 21.4, respectively, *P* = 0.44). However, 1-year mortality rates were higher for NF-LG AS (11.8%) compared to HG-AS (6.2%, *P* = 0.001). Patients with NF-LG AS had higher earlier mortality rates than those with HG-AS, and survival differences remained significant over the first year of follow-up (HR: 2.41, 95% CI: 1.35-4.28). NF-LG AS deaths disproportionately occurred in patients with very poor baseline QoL. There were no significant differences in nonfatal adverse procedure-related events between groups, and so the survival difference was felt to be related to baseline comorbidities rather than procedure-related.

By studying patient QoL, this article provides insight into an important patient-centered question: could more patients benefit from TAVR? While QoL assessments are prone to placebo effect, this study used KCCQ scores in a real-world population undergoing TAVR with similar improvements found in all hemodynamic groups. This study was a retrospective cohort of patients from a single center. Most patients (86.6%) received a self-expanding valve, limiting extrapolation to balloon-expandable valves. Importantly, the patients in this study do not represent all-comers with NF-LG AS but rather those who were selected by a multidisciplinary team to undergo TAVR. Thus, results are vulnerable to unmeasured confounding and selection bias. The higher mortality among those with NF-LG AS compared to HG-AS may reflect unmeasured baseline differences between the 2 groups. How to select patients with NF-LG AS who may benefit from TAVR remains an unanswered question. As the authors acknowledge, NF-LG AS likely represents a heterogeneous group of patients. In some cases, measurement error may account for the discrepancy between AVA and gradients; in others, the discrepancy may be due to concomitant valvular disease, low transvalvular flow rate despite normal SVi, hypertension and reduced arterial compliance, and inherent inconsistencies in the guideline criteria for severe AS.^17^ Additionally, as all patients in the study underwent TAVR, the results do not elucidate the comparative effectiveness of TAVR vs medical management in patients with NF-LG AS. The natural history and prognosis of untreated NF-LG AS require further study. Ultimately, these results add to our incremental knowledge base but do not answer the clinical conundrum of how best to manage our next patient with NF-LG AS.

This study reveals new information about QoL improvements for patients with NF-LG AS undergoing TAVR, a subset of AS patients that remains poorly understood. Still, we must be cautious to avoid indication creep—the tendency to extend therapies to inadequately studied indications. As we consider TAVR for hemodynamic phenotypes where data are sparse or for low-risk patients with longer life expectancies, we must simultaneously acknowledge small but real procedural risks, such as conduction system injury, prosthetic paravalvular leak, jeopardized coronary access, and prosthetic valve deterioration. We can cause serious harm at a population level without holding ourselves to more robust prospective data with longer follow-up. As the authors highlight, we have more to learn about the role of TAVR in patients with NF-LG AS.

## Funding support and author disclosures

Dr McLaughlin has received funding from the UCSF-Bay Area Center for AIDS Research, a National Institutes of Health-funded program (P30 AI027763). Dr Goyal has ownership interest in CardioNerds, which has received funding from American College of Cardiology, American Society for Preventive Cardiology, Patreon donors, Make a Dent organization, Amarin, Amgen, Esperion, Illumina, Pfizer, Zoll LifeVest, Glass Health, Locum Story, Med Mastery, Medscape, Panacea Financial, Novo Nordisk, and Cardiac Dimensions. All other authors have reported that they have no relationships relevant to the contents of this paper to disclose.
